# Regulation of neurons in the dorsal motor nucleus of the vagus by SIRT1

**DOI:** 10.3389/fnins.2013.00270

**Published:** 2014-01-10

**Authors:** Yanyan Jiang, Andrea Zsombok

**Affiliations:** ^1^Neuroscience Program, School of Science and Engineering, Tulane UniversityNew Orleans, LA, USA; ^2^Department of Physiology, School of Medicine, Tulane UniversityNew Orleans, LA, USA

**Keywords:** resveratrol, SIRT1, ATP-sensitive potassium channel, PI3-kinase, dorsal motor nucleus of vagus, patch-clamp

## Abstract

Neurons in the dorsal motor nucleus of the vagus (DMV) play a critical role in the regulation of autonomic functions. Previous studies indicated that central activation of sirtuin 1 (SIRT1) has beneficial effects on homeostasis, most likely via modulation of the autonomic output. Sirtuins are NAD^+^-dependent deacetylases and have been associated with longevity. SIRT1 is one of the best-characterized sirtuins expressed in mammals, and may be involved in the regulation of metabolism. Resveratrol, a SIRT1 activator reduced hyperglycemia likely through activation of vagal output; however, the cellular mechanisms of action have not been determined. In this study, whole-cell patch-clamp electrophysiology on acute brainstem slices was used to test the hypothesis that activation of SIRT1 with resveratrol enhances neurotransmission in DMV neurons. Application of resveratrol increased the frequency of spontaneous excitatory postsynaptic currents (sEPSC). This effect was K_ATP_ channel-dependent and was prevented with pre-application of SIRT1 inhibitor, EX527. Resveratrol also increased miniature EPSC (mEPSC) frequency without change in amplitude. Furthermore, our data demonstrated that resveratrol regulates excitatory neurotransmission in a PI3 kinase-dependent manner, since wortmannin, a PI3K inhibitor prevented the increase of mEPSC frequency caused by resveratrol. In conclusion, our data demonstrate that resveratrol via SIRT1 increases excitatory neurotransmission to DMV neurons. These observations suggest that activation of SIRT1 may regulate the function of subdiaphragmatic organs through controlling the activity of parasympathetic DMV neurons.

## Introduction

Neurons within the dorsal motor nucleus of the vagus (DMV) are parasympathetic motor neurons as they project to the periphery and regulate the tone to most of the subdiaphragmatic organs and thus, regulate feeding, digestion, energy, and glucose homeostasis (Laughton and Powley, 1987; Berthoud, 2008). The activity of DMV neurons is largely controlled by local circuits and by inputs from other brain regions including the hypothalamus (Saper et al., 1976; Swanson and Sawchenko, 1980; Zsombok and Smith, 2009). Hormones, metabolic signals, gastrointestinal signals, or pharmacological agents have the potential to alter the activity of DMV neurons and thereby modulate the parasympathetic outflow to the organs. Therefore, there is a continuous search to identify potential therapeutic agents that alter synaptic activity and thus, influence the function of the visceral organs.

Sirtuins are NAD^+^-dependent histone deacetylases that are highly conserved throughout the evolution (Imai et al., 2000; Michan and Sinclair, 2007). Sirtuins play protective roles promoting the survival of the organism and it has been suggested that they may serve as the molecular link between calorie restriction and prolonged lifespan following dietary restrictions (Cohen et al., 2004; Michan and Sinclair, 2007; Haigis and Sinclair, 2010; Satoh et al., 2010; Coppari, 2012). Sirtuin 1 (SIRT1) is one of the best characterized sirtuins and plays a pivotal role in adaptive responses to high-energy states and hypercaloric diets (Haigis and Sinclair, 2010). Liver-specific deletion of SIRT1 impairs lipid metabolism and reduces glucose production (Erion et al., 2009; Purushotham et al., 2009). In pancreatic beta cells, SIRT1 increases insulin secretion through reduction of uncoupling protein 2 (UCP2) (Moynihan et al., 2005). In addition to its peripheral action, activation of SIRT1 in the brain improves diet-induced diabetes (Ramadori et al., 2009). Central administration of resveratrol, a SIRT1 activator can normalize diet-induced hyperglycemia and mediate anti-diabetic actions (Ramadori et al., 2009). On the other hand, fasting increases SIRT1 levels in the hypothalamus, and blockade of SIRT1 in the hypothalamus decreases food intake and body weight (Ramadori et al., 2008; Cakir et al., 2009). Resveratrol administration into the hypothalamus also improved insulin sensitivity and hepatic vagotomy significantly attenuated this effect (Knight et al., 2011). Despite that the expression of SIRT1 has been shown in brain areas involved in energy and glucose homeostasis, including the hypothalamus and the dorsal vagal complex of the brainstem (Ramadori et al., 2008) and the *in vivo* studies suggested its beneficial effects, the synaptic mechanism underlying the actions of resveratrol in the brain remained to be determined.

In this study, we used whole-cell patch-clamp electrophysiology from DMV neurons to test the hypothesis that activation of SIRT1 with resveratrol enhances neurotransmission in DMV neurons. Our data demonstrate that resveratrol increased spontaneous and miniature excitatory neurotransmission through modulation of ATP-sensitive K^+^ channels (K_ATP_) in a PI3-kinase-dependent manner.

## Methods

### Animals

Male CD1 mice (6–8 weeks; Harlan Laboratories, Indianapolis, IN) were used for these experiments. Animals were housed in a vivarium under 12-h light, 12-h dark cycle with food and water available *ad libitum*. Experiments were performed under the guideline of National Institute of Health Guide for the Care and Use of Laboratory Animals and approved by Tulane University's Institutional Animal Care and Use Committee.

### Brain slices preparation

Acute brainstem slices containing the DMV were prepared as previously described (Williams et al., 2007; Zsombok et al., 2011). Under deep anesthesia, mice were decapitated and the brain was removed and immersed in ice-cold oxygenated artificial cerebrospinal fluid (aCSF) containing the following (in mM): 124 NaCl, 26 NaHCO_3_, 1.4 NaH_2_PO_4_, 11 glucose, 3 KCl, 1.3 MgCl_2_, 1.5 CaCl_2_, pH 7.3–7.4. Transverse brainstem slices (300 μm) were cut with a vibratome (Leica), and then the slices were transferred to a holding chamber containing aCSF (34–36°C, ~1 h) before being transferred to a recording chamber mounted on a fixed stage under an upright microscope (Nikon FN1).

### Whole-cell patch-clamp recordings

Whole-cell patch-clamp recordings were performed at 34–36°C. Neurons were identified under 40x water-immersion objective (N.A = 0.8) using infrared illumination and differential interference contrast optics (IR-DIC). For whole-cell patch-clamp recordings, electrodes (2–5 MΩ) were filled with a solution containing the following (in mM): 130 K^+^ gluconate, 10 HEPES, 5 EGTA, 1 NaCl, 1 MgCl_2_, 1 CaCl_2_, 3 KOH, 2–3 Mg-ATP, pH 7.3–7.4. Excitatory postsynaptic currents (EPSCs) were examined at a holding potential of −60 mV. Electrophysiological signals were recorded using an Axoclamp 700B amplifier (Molecular Devices) and acquired by pClamp 10 (Molecular Devices). Synaptic currents were analyzed offline using pClamp 10 and MiniAnalysis (Synaptosoft).

### Drug application

Tetrodotoxin (TTX, 1 μ M, Tocris Bioscience) was used in the bath solution in specific experiments to block action potential and monitor miniature EPSCs (mEPSCs). The SIRT1 activator resveratrol (1–500 μ M, Tocris Bioscience) and the selective SIRT1 inhibitor EX527 (500 nM, Tocris Bioscience) were dissolved in ethanol and diluted in aCSF (final concentration of ethanol <0.1% by volume). The ATP-sensitive K^+^ channel blocker glibenclamide (1 μ M, Tocris Bioscience), and a PI3-kinase inhibitor wortmannin (1 μ M, Tocris Bioscience) were dissolved in DMSO and diluted in aCSF (final DMSO concentration <0.01%).

### Statistical analysis

Continuous recordings of EPSCs have been conducted before and after application of the drugs and the data were analyzed in 2 min epochs. We observed the maximum effect of resveratrol ~6–8 min following bath application and we have used this time point in bar-graphs. The effect of activators and inhibitors on spontaneous and mEPSC frequency and amplitude were analyzed within individual cells using the Kolmogorov-Smirnov test by comparing 2 min epochs before and 6–8 min after drug application. The effects of drug applications across the neuron groups were analyzed using a paired two-tailed Student's *t*-test. For all analysis, probability values over the 95% confidence level (*p* < 0.05) were considered significant. Numbers were reported as mean ± standard error (SEM).

## Results

### Resveratrol increased spontaneous excitatory neurotransmission

**Figure 1:**
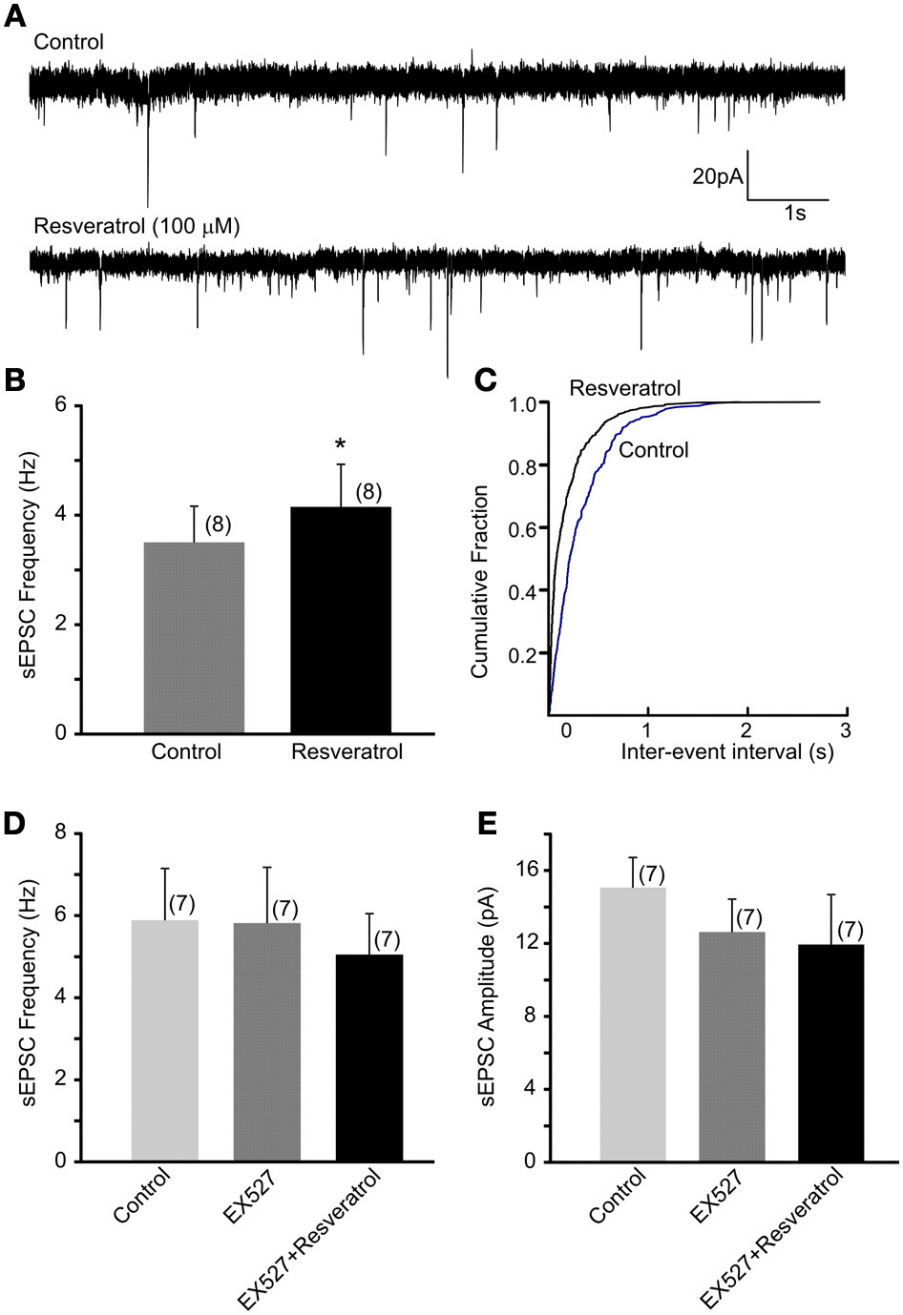
**Resveratrol increased spontaneous excitatory neurotransmission through SIRT1 activation in neurons of the dorsal motor nucleus of the vagus (DMV). (A)** Continuous whole-cell patch-clamp recordings of spontaneous EPSCs (sEPSCs) at holding potential of −60 mV before (upper trace) and after (lower trace) resveratrol application. **(B)** Combined data showing increased frequency of sEPSCs following resveratrol application. ^*^Significance (*p* < 0.05). **(C)** Cumulative event probability plot of inter-event interval distribution in the recording shown in **(A)**. **(D)** Mean group data showing that in the presence of a selective SIRT1 inhibitor EX527 resveratrol failed to increase sEPSC frequency in DMV neurons. **(E)** Combined data showing no significant change in amplitude of sEPSCs following resveratrol application in the presence of EX527.

Previous *in vivo* findings revealed that the beneficial effect of central administration of resveratrol is modulated by the autonomic nervous system. Hepatic vagotomy attenuated this effect (Knight et al., 2011) suggesting the involvement of synaptic mechanisms at the level of DMV. Here, we have assessed the excitatory control of DMV neurons following SIRT1 activation with resveratrol. Recordings of spontaneous EPSC (sEPSCs) were conducted at −60 mV. The average frequency of sEPSCs was 3.5 ± 0.7 Hz (range from 0.7 to 6.5 Hz, *n* = 8). After bath application of 100 μM resveratrol the frequency of sEPSCs significantly increased to 4.2 ± 0.8 Hz (range from 0.8 to 8.2 Hz, *n* = 8, *p* < 0.05) (Figures 1A–C). The average amplitude of sEPSCs was 13.2 ± 1.4 pA (range from 7.5 to 19.2 pA) before and 10.3 ± 0.6 pA (range from 8.0 to 13.7 pA) after application of resveratrol (*n* = 8, *p* < 0.05).

To verify that the increased sEPSC frequency is due to SIRT1 activation we pre-incubated the slices with a selective SIRT1 inhibitor, EX527 (500 nM). The average frequency of sEPSCs was 5.9 ± 1.3 Hz (range from 1.1 to 10.1 Hz, *n* = 7) before and 5.8 ± 1.3 Hz (range from 0.6 to 11.9 Hz) after application of EX527 indicating no change in sEPSC frequency in the presence of EX527 (*p* > 0.05). Furthermore, application of resveratrol (100 μM) in the presence of EX527 did not increase sEPSC frequency (5.1 ± 1.0 Hz, range from 0.5 to 8.5 Hz, *n* = 7, *p* > 0.05) (Figure 1D). The amplitude of sEPSCs was 15.6 ± 1.7 pA in aCSF, 12.6 ± 1.8 pA in the presence of EX527 and 11.9 ± 2.7 pA after resveratrol application (*n* = 7, *p* > 0.05) (Figure 1E). Together, our data indicate that resveratrol through SIRT1 activation significantly increased spontaneous excitatory neurotransmission in DMV neurons.

### Resveratrol increased miniature excitatory synaptic neurotransmission

**Figure 2:**
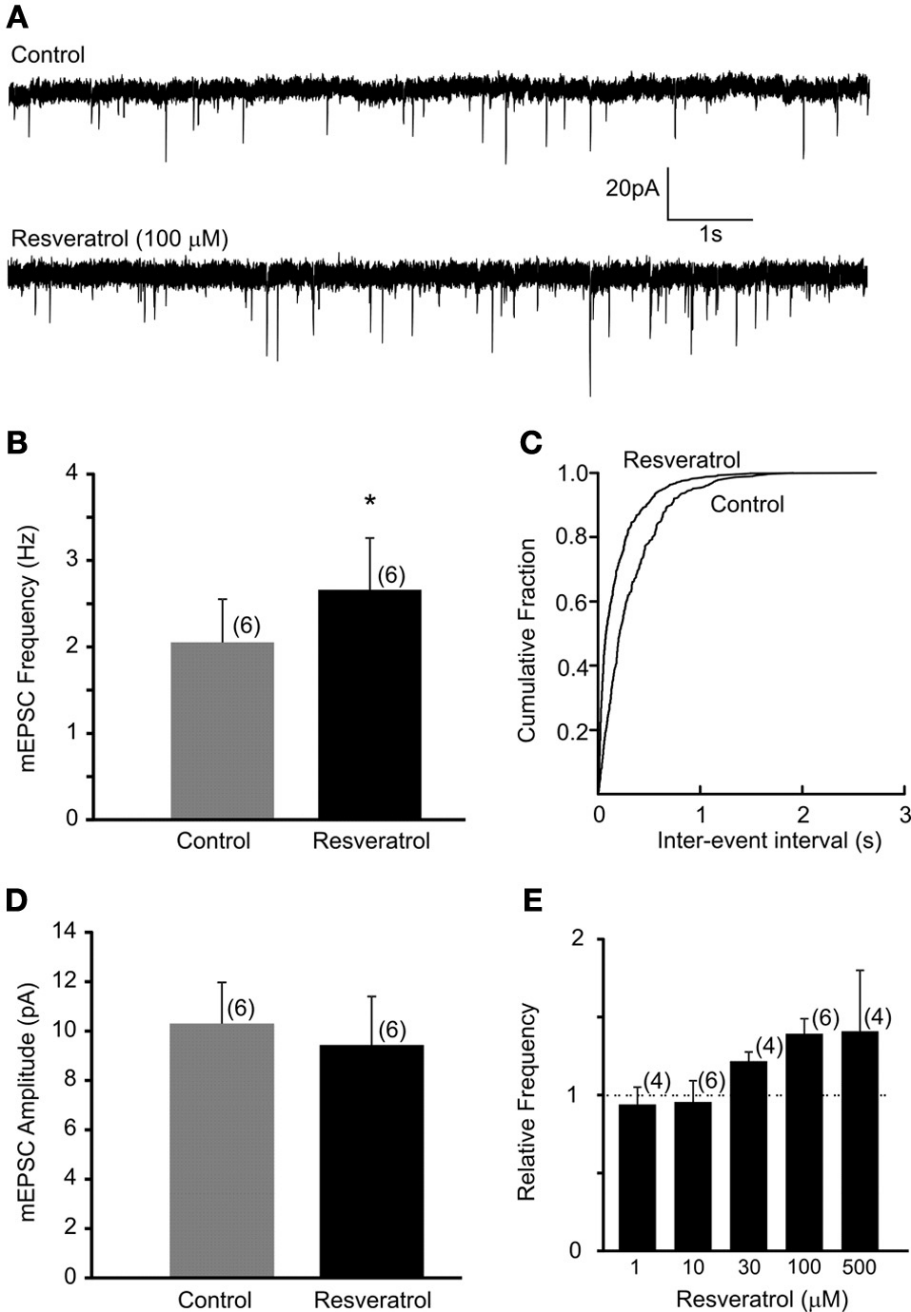
**Resveratrol enhanced miniature excitatory neurotransmission in DMV neurons. (A)** Voltage-clamp recordings of mEPSCs from a DMV neuron before (upper trace) and after (lower trace) resveratrol application. (V_*m*_ = −60 mV). **(B)** Combined data demonstrating that resveratrol increased mEPSC frequency. ^*^Significance (*p* < 0.05). **(C)** Cumulative event probability plot of inter-event interval distribution in the recording shown in **(A)**. **(D)** Mean group data showing that resveratrol did not alter the amplitude of mEPSCs. **(E)** Histogram showing the concentration dependence of the response to resveratrol in DMV neurons. Number of replicates indicated at each concentration in parentheses.

Recordings of miniature EPSCs were conducted in the presence of TTX (1 μM) to block action potential dependent neurotransmitter release. The average frequency of mEPSCs was 2.1 ± 0.5 Hz (range from 0.4 to 3.5 Hz, *n* = 6). Bath application of resveratrol (100 μM) significantly increased the frequency of mEPSCs to 2.7 ± 0.6 Hz (range from 0.6 to 4.6 Hz, *n* = 6, *p* < 0.05) without altering the amplitude (10.3 ± 1.7 vs. 9.4 ± 2.0 pA, *n* = 6, *p* > 0.05) (Figure 2). These data demonstrate that resveratrol via SIRT1 activation increased mEPSC frequency and suggest presynaptic site of action.

Previous studies used resveratrol in a variety of concentrations suggesting that resveratrol may alter synaptic transmission in dose-dependent manner. Therefore, we conducted additional experiments using resveratrol from 1 to 500 μM. Our data demonstrated that resveratrol did not alter the frequency of mEPSCs at concentration of 1 and 10 μM in the recorded DMV neurons (*p* > 0.05) (Figure 2E). Application of 30 μM resveratrol increased the frequency of mEPSCs from 4.0 ± 0.4 (range from 3.3 to 4.5 Hz) to 4.8 ± 0.3 Hz (range from 4.1 to 5.3 Hz, *n* = 4, *p* < 0.05) (Figure 2E). Application of 500 μM of resveratrol increased mEPSC frequency from 2.4 ± 0.8 (range from 0.9 to 3.8 Hz) to 3.3 ± 0.6 Hz (range from 2.2 to 4.4 Hz, *n* = 4) (Figure 2E).

### Mechanism of effect on synaptic transmission

**Figure 3:**
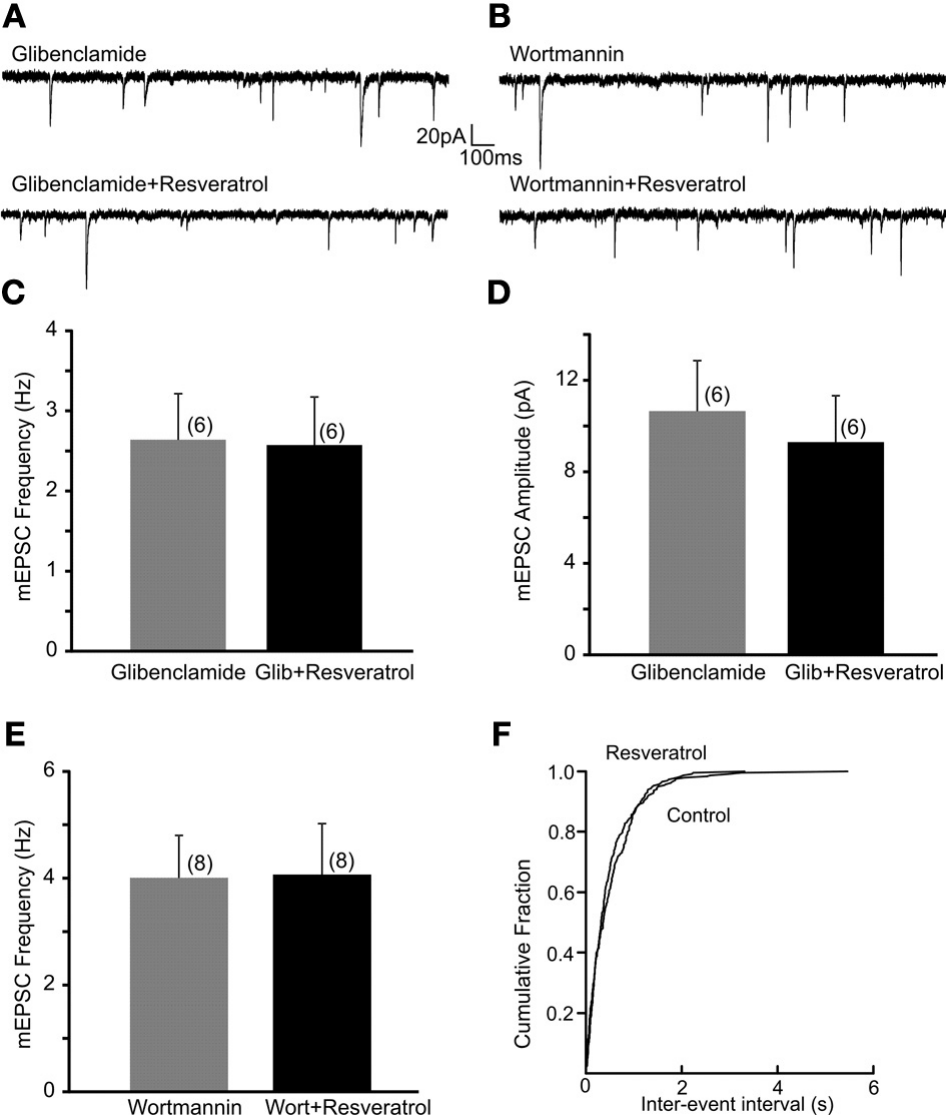
**Resveratrol increased excitatory neurotransmission through PI3-kinase activated K_ATP_ channels. (A)** Continuous whole-cell patch-clamp recordings of mEPSCs before (upper trace) and after (lower trace) resveratrol (100 μM) application in the presence of glibenclamide (1 μM). **(B)** Voltage-clamp recordings showing mEPSCs recordings before (upper trace) and after (lower trace) resveratrol application in the presence of wortmannin (1 μM). **(C)** Combined data demonstrating that resveratrol failed to increase mEPSC frequency in the presence of glibenclamide. **(D)** Combined data indicating no change in amplitude of mEPSCs after resveratrol application in the presence of glibenclamide. **(E)** Combined data showing that resveratrol failed to increase mEPSC frequency in the presence of wortmannin. **(F)** Cumulative event probability plot of inter-event interval distribution in the recording shown in **(B)**.

Previous *in vivo* and cell culture studies suggested that resveratrol may alter ATP-sensitive K^+^ channels (Chen et al., 2007; Knight et al., 2011). To investigate whether K_ATP_ channels are involved in the regulation of excitatory neurotransmission following resveratrol administration we have used a K_ATP_ channel blocker glibenclamide and investigated its effect on the resveratrol induced increase of EPSC frequency. Slices were incubated in aCSF containing glibenclamide (1 μM) for 30 min to block K_ATP_ channels, and then recordings were conducted. The average frequency of spontaneous EPSCs in the presence of glibenclamide (1 μM) was 3.7 ± 0.7 Hz (range from 1.1 to 7.9 Hz, *n* = 9). Application of resveratrol (100 μM) in the presence of glibenclamide did not increase sEPSC frequency (3.9 ± 0.7 Hz, *n* = 9, *p* > 0.05; not shown). The average frequency of mEPSCs in the presence of glibenclamide was 2.6 ± 0.6 Hz (range from 0.9 to 4.4 Hz, *n* = 6). In the presence of glibenclamide application of resveratrol failed to increase mEPSC frequency (2.6 ± 0.6 Hz, range from 0.9 to 4.9 Hz, *n* = 6, *p* > 0.05) (Figures 3A,C). Resveratrol did not have effect on the amplitude of mEPSCs (10.6 ± 2.2 vs. 9.3 ± 2.0 pA, *p* > 0.05) (Figure 3D). Our data suggest that resveratrol acts to increase synaptic transmission through K_ATP_ channels. In addition, we have conducted experiments to determine the effect of glibenclamide alone on mEPSC frequency. The average frequency of mEPSCs was 3.9 ± 0.5 Hz (range from 2.0 to 6.2 Hz) before and 4.8 ± 0.7 Hz (range from 2.9 to 7.2 Hz; *n* = 6; *p* < 0.05) after application of glibenclamide. These data demonstrate the modulation of excitatory neurotransmission by K_ATP_ channel.

Next, DMV neurons were exposed to wortmannin, a PI3-kinase inhibitor for 30 min to determine whether the resveratrol induced increase of excitatory neurotransmission is PI3-kinase dependent. Slices were perfused with wortmannin (1 μM) and mEPSCs were recorded. The average frequency of mEPSCs in the presence of wortmannin was 4.0 ± 0.8 Hz (range from 1.9 to 7.8 Hz, *n* = 8) while 4.1 ± 1.0 Hz (range from 1.2 to 8.7 Hz) after resveratrol application (*p* > 0.05) (Figures 3B,E,F). These findings demonstrate that the PI3-kinase pathway is involved in the resveratrol induced increase of EPSC frequency. The average amplitude of mEPSCs was 15.9 ± 3.3 (range from 7.2 to 29.5 pA, *n* = 8) and 11.7 ± 2.2 pA (range from 5.5 to 21.2 pA, *p* < 0.05) after resveratrol administration.

### Resveratrol did not alter the membrane potential or input resistance of DMV neurons

Since resveratrol altered synaptic neurotransmission through K_ATP_ channels we determined the effect of resveratrol on membrane potential and input resistance. The resting membrane potential of recorded DMV neurons was −45.9 ± 1.7 mV (range from −39.7 to −50.4 mV, *n* = 6). Application of resveratrol did not result in a significant change of membrane potential (−46.2 ± 1.1 mV, range from −42.3 to −49.5 mV, *n* = 6, *p* > 0.05). The input resistance of DMV neurons was not different before and after resveratrol application (0.9 ± 0.09 vs. 1.1 ± 0.1 GΩ). These data suggest that despite resveratrol effects on K_ATP_ channels, it does not alter membrane potential or input resistance of the recorded DMV neurons, further indicating presynaptic mechanisms.

## Discussion

In this study we present novel evidence for synaptic regulation of DMV neurons by resveratrol. The following major findings have emerged from this investigation: (1) resveratrol increases spontaneous and mEPSC frequency via SIRT1 activation; (2) resveratrol modulates excitatory neurotransmission through PI3-kinase activated K_ATP_ channels; and (3) resveratrol did not alter membrane potential and input resistance, implying presynaptic mechanism of action.

The increasing prevalence of diabetes, obesity and metabolic syndrome results in a need to identify potential therapeutic targets for the management of these devastating diseases. SIRT1 has been considered as a potential therapeutic target for a variety of diseases including metabolic disorders (Haigis and Sinclair, 2010). Resveratrol, a natural compound found in grapes and red wine has been shown as an effective SIRT1 activator (Howitz et al., 2003). Numerous studies conducted on animals demonstrated that resveratrol improves glucose metabolism (Baur and Sinclair, 2006; Baur et al., 2006; Barger et al., 2008; Andersen et al., 2011; Kang et al., 2012; Marchal et al., 2012), reduces inflammation (Rivera et al., 2009), reverses non-alcoholic fatty liver disease (Bujanda et al., 2008) and prevents obesity (Dal-Pan et al., 2010). On the other hand the results of clinical studies using resveratrol are controversial. It has been reported that oral administration of resveratrol improves mean hemoglobin A1C, systolic blood pressure, total cholesterol, and protein in type 2 diabetic patients (Bhatt et al., 2012). In contrast, a more recent study using high-dose resveratrol supplementation in obese men reported no effect on endogenous glucose production, blood pressure, energy expenditure, fat mass or inflammatory and metabolic biomarkers (Poulsen et al., 2013) raising debates about the effectiveness of resveratrol as a dietary supplement in humans; however, additional studies with more subjects would be necessary to make conclusion.

In addition to the overall effect of resveratrol, the question regarding peripheral or central mechanisms of resveratrol also has been investigated. SIRT1 protein expression has been detected with Western blot analysis in the rat hypothalamus markedly in the arcuate, ventromedial, dorsomedial hypothalamus, and paraventricular nucleus (Knight et al., 2011). This is consistent with *in situ* hybridization and immunohistochemical studies performed on mice that showed marked expression of SIRT1 mRNA and protein in metabolically relevant brain areas including the above mentioned sites (Ramadori et al., 2008; Sasaki et al., 2010). The Ramadori et al. study also demonstrated high expression in the brainstem including the area postrema and the nucleus of the solitary tract (NTS) suggesting SIRT1 expression throughout the neuroaxis involved in energy and glucose homeostasis (Ramadori et al., 2008). Administration of resveratrol into the brain improved insulin sensitivity and normalized hyperglycemia (Ramadori et al., 2009; Knight et al., 2011); however, the underlying synaptic mechanisms are not fully understood. Our study demonstrated that resveratrol, a SIRT1 activator increased excitatory neurotransmission to parasympathetic DMV neurons. Our findings also revealed that the resveratrol effect requires the involvement of K_ATP_ channel and the PI3-kinase pathway.

Glutamate, the main excitatory neurotransmitter is released in the DMV from inputs arriving from many different brain areas including the hypothalamus and the NTS (Travagli et al., 1991; Jiang et al., 2003; Davis et al., 2004). *In vitro* application of resveratrol increased the frequency of both spontaneous and mEPSCs in the DMV indicating a presynaptic action of resveratrol. Our observations also revealed that resveratrol alters EPSC frequency of DMV neurons in dose-dependent manner. The resveratrol induced frequency increase of EPSCs was prevented in the presence of a SIRT1 inhibitor EX527. EX527 is a selective inhibitor of SIRT1 that does not inhibit other sirtuins or histone deacethylase, therefore, our data verified that resveratrol exerts its effect through SIRT1 activation.

**Figure 4:**
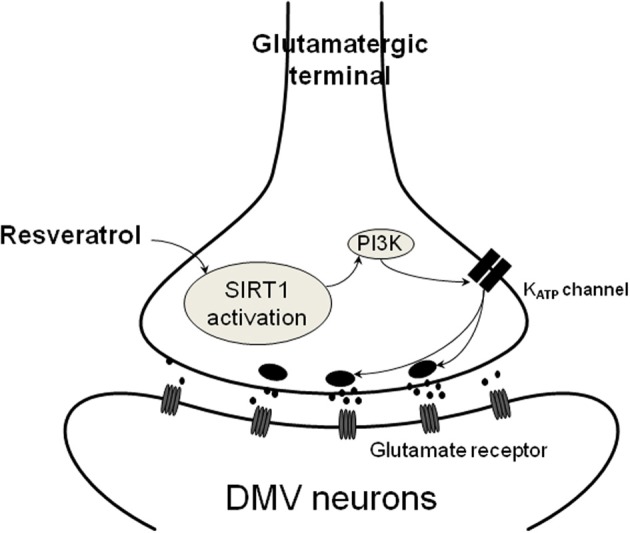
**Schematic illustration of resveratrol action on presynaptic terminals**. SIRT1 activation with resveratrol results in increased glutamate release from presynaptic terminals. This mechanism depends on PI3-kinase dependent closure of K_ATP_ channels leading to depolarization and increased neurotransmitter release.

Previous studies established that resveratrol alters ion channels by various mechanisms depending on the cell types. Resveratrol activates BK channels in endothelial cells (Li et al., 2000), inhibits I_K_ channels in cultured rat hippocampal neurons (Gao et al., 2006; Dong et al., 2013) and inhibits TRP channels in HEK and dorsal root ganglia cells (Yu et al., 2013). The involvement of K_ATP_ channels also has been demonstrated (Chen et al., 2007). This study by Chen revealed that resveratrol significantly inhibits K_ATP_ channels and voltage-gated K^+^ currents in order to depolarize the membrane and increase insulin secretion from pancreatic beta cells. Similarly, the *in vivo* work of Knight and co-workers indicated that the effect of resveratrol was inhibited in the presence of a K_ATP_ channel blocker, glibenclamide (Knight et al., 2011). Our data also suggest that resveratrol increases excitatory neurotransmission in the DMV through K_ATP_ channels (Figure 4). Pre-incubation of the brainstem slices with glibenclamide prevented the resveratrol-dependent increase of mEPSC frequency, suggesting the involvement of K_ATP_ channels. Furthermore, application of glibenclamide alone resulted in an increase of mEPSC frequency, indicating that blocking K_ATP_ channels modulates pre-synaptic neurotransmitter release. K_ATP_ channels have been shown to modulate synaptic neurotransmission in the brainstem (Ferreira et al., 2001; Williams and Smith, 2006; Williams et al., 2007). Previous electrophysiological studies demonstrated that tolbutamide, another K_ATP_ channel blocker depolarizes NTS neurons, suggesting the presence of K_ATP_ channels in brainstem NTS neurons (Williams and Smith, 2006). Depolarization of NTS neurons could lead to increased neurotransmitter release to DMV neurons. Furthermore, the effect of K_ATP_ channel opener diazoxide alone has been shown to reduce mEPSC frequency in DMV neurons, demonstrating that K_ATP_ channels are able to modulate glutamate release at the presynaptic terminals (Williams et al., 2007). Another cellular observation demonstrated that glibenclamide mimics the actions of elevated glucose levels on the amplitude of evoked PSCs in DMV neurons, while diazoxide, a K_ATP_ opener had opposite effect (Ferreira et al., 2001). These experiments also suggest a presynaptic site of action and indicate modulation of neurotransmission by K_ATP_ channels.

The involvement of PI3K in K_ATP_-dependent mechanisms is known. Furthermore, it also has been demonstrated that PI3K is involved in SIRT1 activation (Frojdo et al., 2011). Therefore, we also pre-incubated the slices with wortmannin then applied resveratrol and found that wortmannin blunted the resveratrol-dependent increase of EPSC frequency. Previous electrophysiological studies used wortmannin from 10 nM to 3 μM concentration (Williams et al., 2007; Gao et al., 2012). In our experiments we used 1 μM of wortmannin for 30 min, which diminished the effect of resveratrol. It has been shown that this concentration of wortmannin inhibits the mammalian target of rapamycin (mTOR) (Brunn et al., 1996), therefore, there is a possibility that in addition to PI3K, the mTOR signaling is modulated by resveratrol. However, this scenario is unlikely, because a recent study demonstrated that less than 72 h treatment with rapamycin, a specific inhibitor of mTOR did not alter the electrophysiological properties of neurons (Weston et al., 2012). It also has been demonstrated that 3-week long inhibition of mTOR with rapamycin increases the excitability of hypothalamic neurons via K_ATP_ channel (Yang et al., 2012), suggesting that changing the electrophysiological properties of neurons via the mTOR signaling requires longer duration. In our experiments resveratrol increased EPSC frequency within 10 min, therefore, it is unlikely that the observed electrophysiological response involves the mTOR signaling; however, additional studies would be necessary to make conclusion regarding the interaction between resveratrol and mTOR signaling. It has been observed in hypothalamic cells that the presence of PI3K inhibitor *per se* reduced the phosphorylation of protein kinase B (PKB) and glycogen synthase kinase 3 (GSK3) levels, indicating that PI3K is active to a limited degree in neurons of the arcuate nucleus, and it also prevented the leptin and insulin induced phosphorylation of PKB and GSK3 (Mirshamsi et al., 2004). Furthermore, inhibiting PI3K has been shown to decrease insulin-stimulated phosphorylation of MAPK (Mirshamsi et al., 2004), and blocking PI3K can also inhibit insulin induced increase of MAPK activity in adipose tissue (Sajan et al., 1999). It also has been described that inhibition of PI3K reduced the effect of leptin or insulin on K_ATP_ channels (Mirshamsi et al., 2004). Moreover, inhibition of PI3K prevented the leptin caused increase of phosphatidylinositol 3,4,5-triphosphate. PtdIns activates K_ATP_ channels, probably not through direct binding (Harvey et al., 2000; Mirshamsi et al., 2004), but actin remodeling. Furthermore, direct interplay between SIRT1 and insulin signaling pathway including PI3K has been demonstrated in muscle (Frojdo et al., 2011). Downregulation of SIRT1 expression levels diminished insulin-stimulated PKB phosphorylation and overexpression increased insulin-stimulated PKB phosphorylation. SIRT1 positively modulated the activity of upstream components of insulin pathway and SIRT1 interacted with tyrosin phosphorylated proteins and with the PI3K-p85alpha (Frojdo et al., 2011). In addition, it has been shown that resveratrol also could directly modulate the sulfonylurea receptor 1 (SUR1) of K_ATP_ channels (Hambrock et al., 2007), indicating a possible direct link between resveratrol and binding to SUR1. In summary, the evaluation of interactions among the above mentioned intracellular pathways remains to be determined and could be the subject of future studies.

Together, these data indicate that pre-application of glibenclamide or wortmannin prevented the resveratrol induced increase of EPSC frequency that might imply a PI3-kinase activated K_ATP_ channel in response. Previous data demonstrated that the resveratrol effect depends on K_ATP_ channels and hepatic vagotomy significantly attenuated this effect (Knight et al., 2011), indicating the involvement of the parasympathetic nervous system. Our data confirmed that resveratrol is able to modulate neurotransmission to DMV neurons. The synaptic effects of resveratrol appear to be due to increased glutamate release, likely via closing K_ATP_ channels, from synaptic terminals contacting DMV neurons (Figure 4). Based on the high expression of SIRT1 mRNA detected in the NTS, one possible origin of the synaptic terminals is the NTS. Another possibility is the hypothalamus (Ramadori et al., 2008; Sasaki et al., 2010). Both brain areas are known to send projections to the DMV (Swanson and Sawchenko, 1980; Travagli et al., 1991) and thereby involved in the modulation of parasympathetic output to the subdiaphragmatic organs.

Our findings identified a potential cellular mechanism underlying the effect of resveratrol administration into the brain. Considering the described anti-diabetic effects of central resveratrol administration and that vagotomy attenuated this effect (Ramadori et al., 2009; Knight et al., 2011) we can speculate that modulating the synaptic activity of DMV neurons underlies the effect of resveratrol on the autonomic nervous system.

## Author contributions

Yanyan Jiang conducted experiments and contributed to the manuscripts preparation; Andrea Zsombok designed the experiments and prepared the manuscript.

### Conflict of interest statement

The authors declare that the research was conducted in the absence of any commercial or financial relationships that could be construed as a potential conflict of interest.
